# The Impact of Storage Conditions on DNA Preservation in Human Skeletal Remains: A Comparison of Freshly Excavated Samples and Those Stored for 12 Years in a Museum Depot

**DOI:** 10.3390/genes16010078

**Published:** 2025-01-11

**Authors:** Tonja Jeromelj, Tamara Leskovar, Irena Zupanič Pajnič

**Affiliations:** 1 Institute of Forensic Medicine, Faculty of Medicine, University of Ljubljana, Korytkova 2, 1000 Ljubljana, Slovenia; tjeromelj@gmail.com; 2 Centre for Interdisciplinary Research in Archaeology, Department of Archaeology, Faculty of Arts, University of Ljubljana, 1000 Ljubljana, Slovenia; tamara.leskovar@ff.uni-lj.si

**Keywords:** storage conditions, DNA preservation, petrous bone, long-term storage, DNA yield, DNA degradation

## Abstract

**Background:** As the field of ancient DNA research continues to evolve and produce significant discoveries, it is important to address the crucial limitations it still faces. Under conducive conditions, DNA can persist for thousands of years within human skeletal remains, but, as excavation occurs, the environment abruptly changes, often leading to the loss of DNA and valuable genetic information. Proper storage procedures are needed to mediate DNA degradation and maintain sample integrity. This study aimed to investigate the impact of long-term storage under unregulated temperatures and humidity conditions on DNA preservation in human skeletal remains. **Methods:** To achieve this, archaeological petrous bones were used for DNA recovery. The DNA yield and degree of DNA degradation were compared for samples originating from historically and geographically equivalent archaeological sites, which differed in times of excavation and, consequently, in storage durations and conditions. DNA yield and the degree of DNA degradation were determined using real time PCR. **Results:** A significant reduction in the DNA yield and a borderline significant increase in the degree of DNA degradation were detected for samples stored at unregulated conditions for approximately 12 years. **Conclusions:** Our results show the imperative need for adhering to scientific recommendations regarding the optimal temperature and humidity in the long-term storage of human skeletal material.

## 1. Introduction

Obtaining sufficient amounts of high-quality ancient DNA (aDNA) from human skeletons remains a significant challenge in forensic and archaeological genetics. Due to its inherent susceptibility to degradation, fragmentation, and exogenous contamination [1], the optimal preservation of skeletal remains is critical for ensuring the integrity of genetic material. Although numerous studies and reviews have addressed DNA extraction protocols from skeletal material [2,3,4], relatively little research has focused on the optimal storage conditions for human skeletal remains to preserve their integrity and prevent information loss over time.

Skeletal remains, along with the DNA they harbor, begin to decay immediately after death [5,6]. Like any material, skeletal remains function as an open system, exchanging energy and substances with their environment due to differences in composition. These interactions lead to energy instability and an imbalance between the material and its surroundings. To achieve stability, the chemical and structural properties of the material change. As equilibrium is approached, these changes diminish, eventually stabilizing the material for long-term preservation. However, this equilibrium can be disrupted by environmental changes—such as abrupt shifts during excavation or fluctuating storage conditions—or may never fully establish itself. In the absence of stability, continuous changes persist, eventually resulting in complete decay [7]. Therefore, storing skeletal remains in a stable environment is essential for long-term preservation.

The decay of skeletal remains is strongly influenced by environmental factors such as temperature, humidity, and pH [5,6]. Current guidelines for storage recommend maintaining a maximum temperature of 16–20 °C and relative humidity between 45% and 65% while avoiding significant fluctuations [8,9,10]. However, many museums are unable to adhere to these guidelines due to the size of their collections, limited storage space, and financial constraints. Furthermore, systematic research on the subject is lacking [5], leaving uncertainty about the optimal storage conditions for preserving human skeletal remains and their DNA. The consequences of storing these remains under unregulated conditions also remain poorly understood.

This study investigates the impact of long-term storage under unregulated temperature and humidity conditions on DNA preservation in human skeletal remains. By comparing the quantity and integrity of DNA from freshly excavated skeletal remains with those stored for 12 years under seasonal fluctuations in temperature and humidity, this research aims to underscore the critical role of storage conditions in preserving the molecular integrity of human remains.

## 2. Materials and Methods

### 2.1. Description of Ljubljana’s Archaeological Sites Vrazov Trg and Njegoševa Ulica and Selection of Bone Samples

Archaeological excavations at Ljubljana’s Vrazov trg campus began during the construction of new facilities for the Faculty of Medicine, as the site is located within a registered cultural heritage area. Results of preceding archaeological research at the site revealed the presence of cemetery remains in the courtyard of the building complex, dating back to the modern era. The archaeological exhumations were conducted from 1 June to 20 July of 2023 and uncovered 196 graves. The discovered cemetery is located on the southern side of the Church of St. Peter, the oldest church in the Diocese of Ljubljana, first mentioned in historical sources in 1163 [11]. According to published data and previous exhumations conducted on the northern part of the church (Njegoševa archaeological site), the cemetery dates back to the 9th Century. The cemetery was active throughout the entire Middle Ages and into the modern era until the decree of Maria Theresa in 1784 and the subsequent decree of Joseph II in 1787, which mandated that cemeteries around churches be abolished. Based on stratigraphic evidence, the cemetery can be divided into multiple phases, with most of the grave pits attributed to the earliest phase, having been dug into a layer of humus [12].

Ljubljana’s Njegoševa archaeological site is located on the northern site of The Church of St. Peter in close proximity to the archaeological site of Vrazov trg. The archaeological excavations in the area began in 2011 and ended in 2012. A total of 860 burials and only 287 grave pits were revealed, clustered around the church, meaning that more recent graves were probably dug into the older ones. The preliminary temporal classification of the skeletons was conducted based on the artifacts found in grave pits, stratigraphic evidence, and the arm positions of skeletons. A total of 132 skeletons were classified as early medieval. The burial ground remained active throughout the Middle Ages and expanded considerably in the Modern Age, between the 16th and 18th century, when the number of burials increased significantly. More than 600 skeletons, excavated at the site, were identified as belonging to the Modern Age [13].

At both archaeological sites, most skeletons were oriented along the E–W axis with the head facing west. They were placed in simple wooden coffins, indicative of a typical Christian burial practice. During the exhumations, the skeletons were categorized, photographed, and assigned a number that was retained throughout the entire process.

Ljubljana lies in the central region of Slovenia and experiences a continental climate with annual average temperatures (1948–2019) ranging between 8.6 and 12.6 °C. The absolute highest and lowest temperatures of the year are 40.2 °C and −23.3 °C, respectively (https://meteo.arso.gov.si/met/sl/climate/diagrams/ljubljana/, accessed on 10 October 2024). The excavation site is located in the city center of Ljubljana and geologically belongs to the central part of the Ljubljana Basin.

A total of 38 skeletons from the archaeological site of Vrazov trg were selected for comparison with genetic data of 101 samples from Ljubljana’s Njegoševa site. To establish DNA preservation, only skeletons with preserved petrous bones were chosen. Genetic data for petrous bone samples from Ljubljana’s Njegoševa site were obtained in 2023, following genetic processing after 12 years of storage. The characteristics of bone samples are shown in Supplementary Material S1—SM S1.

Consent for sampling the skeletons from the archaeological site of Ljubljana’s Vrazov trg was obtained from the Museums and Galeries of Ljubljana (MGML). The research received approval from the National Medical Ethics Committee of the Republic of Slovenia (0120-308/2024-2711-3), the ethical approval date is 18 July 2024.

### 2.2. Storage of Bone Samples

During anthropological analysis, skeletons were categorized and assigned a number that remained through the entire process. In the case of Ljubljana’s Vrazov trg, the bones were superficially cleaned of soil. Exhumations at the Vrazov trg site in Ljubljana were completed in July 2023, after which all dry-cleaned bone samples were delivered to our laboratory. The bone samples from the Njegoševa site were excavated in 2011 and were cleaned of soil at the time of excavation. After drying, skeletal remains were stored inside cardboard boxes in a museum depot in Ljubljana for approximately 12 years. The depot is not insulated so the environmental conditions affecting the preservation of samples fluctuate with the weather. The humidity and temperature in the storage room are not measured, but the latter is estimated to range between 5 °C and 35 °C.

### 2.3. DNA Extraction

Petrous bones were sampled from the skeletons. Before cutting, all bones were chemically cleaned with 5% Alconox (Sigma-Aldrich, St. Louis, MO, USA), sterile bi-distilled water (Sigma-Aldrich), and 80% ethanol (Merck, Rahway, NJ, USA) to reduce surface contamination. Petrous bones that were still connected to the rest of the temporal bone were separated from it using a sterilized diamond bone saw (Schick, Schemmerhofen, Germany). The dense part of the petrous bone within the optic capsule was detached from the rest of the petrous bone following Pinhasi’s method [14] and later used for extraction. Thin incisions were made on the surface of petrous bones to enhance the grinding process. Immediately before cutting, the bones were cooled with liquid nitrogen to prevent DNA degradation caused by the heat generated during cutting. Cut bone samples were then chemically cleaned again and left to dry overnight. Tools used in cutting and grinding bone samples were cleaned with 6% sodium hypochlorite, sterile bi-distilled water, and 80% ethanol, followed by sterilization with Europa B xp sterilizer (Tecno-Gaz, Parma, Italy) at 134 °C for 45 min and UV irradiation for 20 min using BLX-Multichannel BioLink DNA Crosslinker (Vilber, Collégien, France). To further prevent contamination, all reagents, with the exclusion of those labeled DNA-free or DNase-free water, bi-distilled water, ethanol, sodium hypochlorite, and laboratory plastics, were sterilized (autoclaved) and disinfected with UV irradiation for 20 min using BLX-Multichannel BioLink DNA Crosslinker (Vilber) before use. The working surfaces were also cleaned with 6% sodium hypochlorite, bi-distilled water, and ethanol and subsequently exposed to UV radiation for 20 min, before and after use.

To ensure the integrity of samples, special measures were followed to avoid contamination of samples with contemporary DNA. Bone preparation and extraction procedures were spatially separated from the post-extraction ones [15]. For the preparation of bone samples, a specifically designed space for handling old skeletal remains within a closed MC3 microbiological safety cabinet (Iskra Pio, Šentjernej, Slovenia), equipped with a HEPA filter and UV light, was used. DNA extraction was performed using 0.5 g of bone powder. Before grinding, the bones were cooled again to prevent the loss of DNA caused by bone overheating. A homogenizer (Bead Beater MillMix 20; Tehtnica, Domel, Železniki, Slovenija) was used to grind bone samples into a fine powder.

The samples were cleaned, ground, decalcified, and purified in accordance with the highly efficient DNA extraction method previously described by Zupanič Pajnič [15].

To control for possible contamination and to monitor the purity of reagents and plastics, extraction-negative controls (ENCs) were included in every batch of samples [16]. To minimize the risk of cross-contamination, no more than 12 bone samples were processed in each extraction batch. Furthermore, an elimination database was formed, using samples of all individuals participating in the study, including personnel involved in excavation, anthropological analysis, and DNA analysis. The sampling consisted of collecting saliva using sterile cotton swabs and extracting DNA from buccal smears. To avoid contaminating bone samples with contemporary DNA, two different machines were used for purifying the elimination database samples and bone samples. A BioRobot EZ1 machine (Qiagen, Hilden, Germany) was used to purify DNA extracted from buccal smears, and an EZ1 Advanced XL machine (Qiagen), exclusively used in our laboratory for purifying DNA from aged bone, was used to purify DNA extracted from bone samples. The EZ1&2 DNA Investigator kit (Qiagen) was used for the purification of bone samples and buccal swabs following the previously published protocol [15] and instructions provided by the manufacturer [17].

### 2.4. DNA Quantification

Real-time PCR (qPCR) analysis was utilized to determine the DNA concentration and its degradation in petrous bones. The short autosomal fragment (85 bp, Auto target), conveying the concentration of nuclear DNA, the Y-chromosomal fragment (Y target), and the long autosomal fragment (294 bp, Deg target), was detected using the PowerQuant System (Promega Corporation, Madison, WI, USA) following the manufacturer’s instructions [18]. Auto and Deg target values were used for calculating the DNA degradation index (Auto/Deg ratio). The PowerQuant System (Promega) also includes an internal PCR control (IPC) to detect the possible presence of inhibitors in the amplification reaction. QuantStudio 5 Real-Time PCR System and Quant-Studio Design and Analysis Software 1.5.1 (Applied Biosystems, Thermo Fischer Scientific, Waltham, MA, USA) were used to export and process the raw data. Auto, Deg, and Y values, Auto/Deg ratio, and IPC shift values, along with their associated standard curves, were determined with the PowerQuant Analysis Tool (https://worldwide.promega.com/resources/tools/powerquant-analysis-tool/, accessed on 15 October 2024). The IPC Shift threshold and the Auto/Deg threshold were set at 0.3 and 2, respectively, as per the manufacturer’s recommendations [18]. The quantity of DNA extracted from 1 g of bone powder was determined for each sample based on the Auto target values. The result was converted into units of ng DNA/g of bone sample. The Auto target values were multiplied by a factor of 100 to account for the use of 0.5 g of bone powder and dilution of the extracted DNA in 50 µL of TE buffer.

### 2.5. Statistical Analysis

A statistical comparison was conducted on bone samples from two archaeologically comparable sites, Ljubljana’s Vrazov trg site, located on the southern side of St. Peter’s Church, and the Njegoševa site, situated on the northern side of the same church. Both sites are historically and geographically equivalent, thus ensuring that all environmental factors affecting DNA preservation, excluding storage of skeletal remains, were as similar as possible.

For the statistical analysis, two parameters were used to describe DNA preservation in bone samples: the Auto target, representing DNA quantity, and the Auto/Deg ratio, representing DNA quality. All parameter values were acquired using the PowerQuant Analysis tool (Promega). Statistical analysis was performed using IBM SPSS Statistics, version 28.0.

To explore the effect of storage of skeletal remains after excavation on DNA preservation, the following research hypotheses were formulated:

Hypothesis 1. There are no statistically significant differences between petrous bones from Njegoševa and Vrazov trg in the amount of DNA extracted (ng DNA/g bone).

Hypothesis 2. There are no statistically significant differences between petrous bones from Njegoševa and Vrazov trg in the degradation ratio (Auto/Deg).

The normality and homogeneity of variance were tested using the Kolmogorov–Smirnov test (with Lilliefors significance correction). The research hypotheses were tested using the 95% confidence intervals for means or medians, as suggested as an appropriate measure for testing the differences among groups, especially in medical studies [19,20,21], using the computer program IBM SPSS Statistics for Windows, version 28.0 (Statistical Package for the Social Sciences Inc., Chicago, IL, USA). As the sample size is relatively small, confidence intervals can have limited power to detect significant differences [21]. Thus, formulated hypotheses were also tested using *p* values. Significance was set as *p* ≤ 0.05.

The database contained data from bone samples of 139 individuals, including 101 petrous bones from the Njegoševa site and 38 from the Vrazov trg site.

Kolmogorov–Smirnov test showed that data are not normally distributed. Thus, non-parametric tests were performed, and medians were used for the confidence intervals.

## 3. Results

### 3.1. DNA Quantification

Results obtained with PowerQuant System (Promega) including bone sample characteristics and parameters of DNA quality and quantity, such as the Auto/Deg ratio, IPC Shift, Auto, Deg, and Y target, are shown in SM S1 (Tables S1 and S2). Values for Auto, Deg, and Y targets are conveyed in units of ng DNA/μL of extract. Additionally, the DNA yield was calculated and expressed in ng DNA/g of bone. To minimize the error and variability in samples, all amplification reactions were performed in duplicates, and the duplicate average was used as the basis for all subsequent calculations.

According to developmental validation by Ewing et al. [22], 0.5 pg of DNA per μL of extract is the minimum concentration recommended for reliable quantification with the PowerQuant qPCR kit (Promega). More than 0.5 pg DNA/μL of extract was detected in all bone samples apart from one, excavated from Ljubljana’s Vrazov trg archaeological site (see SM S1, Table S1, labeled red). DNA degradation varied between bone samples and was highest in samples originating from the Njegoševa archaeological site, with Auto/Deg values reaching 387.70 and an average of 71.84. In contrast, samples from the site of Vrazov trg showed lower degradation levels, with maximum Auto/Deg values of 332.57, with an average of 51.68. The presence of inhibitors was detected in nine samples when the IPC Shift value met or exceeded the IPC Shift threshold value of 0.3 (see SM S1, Tables S1 and S2, labeled red). In the remaining samples, the IPC value was below 0.3, indicating that purification with magnetic bead technology in the EZ1&2 DNA Investigator Kit (Qiagen) was highly efficient. In most ENC samples, no PowerQuant targets were detected. In cases where an amplification product was detected, the amount of DNA did not exceed the detection limit for the PowerQuant kit, showing no contamination issues.

### 3.2. Statistical Analysis

Statistical analysis was performed to determine possible variations in the DNA yield and degree of DNA degradation for samples originating from historically and geographically equivalent archaeological sites—Ljubljana’s Vrazov trg and Njegoševa sites—which differed in times of excavation and, consequently, in storage durations and conditions.

**Figure 1:**
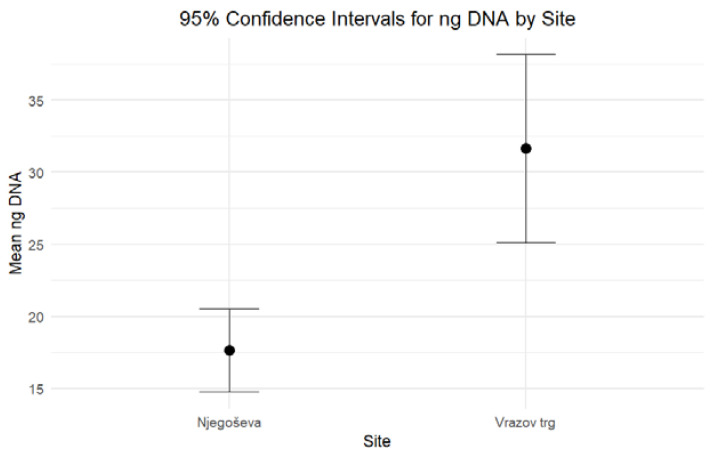
Ninety-five percent confidence intervals for medians for the DNA yield (expressed in ng DNA/g bone) of petrous bones excavated from Njegoševa and Vrazov trg.

**Figure 2:**
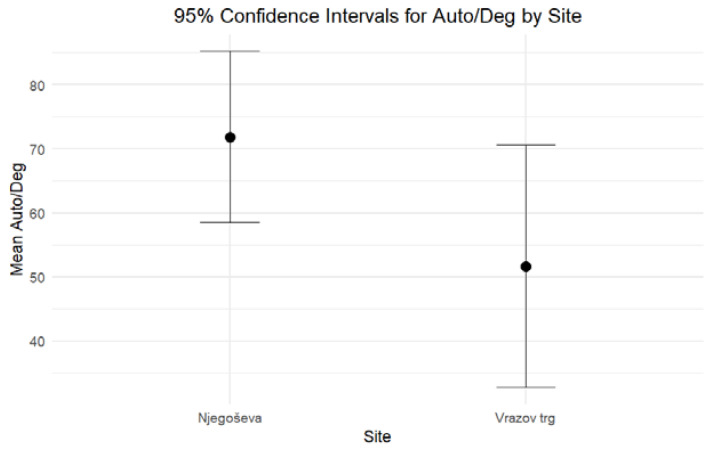
Ninety-five percent confidence intervals for medians for the Auto/Deg ratio of DNA extracted from petrous bones excavated from Njegoševa and Vrazov trg.

When comparing the DNA yield and the degree of DNA degradation for petrous bones excavated from Ljubljana’s archaeological sites of Vrazov trg and Njegoševa, two hypotheses were tested to evaluate whether the two parameters exhibit any statistically significant differences. Based on the combined results of the independent-sample median test and confidence intervals, both formulated hypotheses should be rejected. The results of nonparametric statistic testing showed significant differences (*p* < 0.001) in the DNA yield, while the differences in the Auto/Deg ratio were borderline significant (*p* = 0.053). Petrous bones from Vrazov trg yielded more DNA (Mean = 31.64, Standard Error = 3.22) compared to those from Njegoševa (Mean = 17.66, Standard Error = 1.44) (Figure 1). The Auto/Deg ratio was higher in petrous bones from Njegoševa (Mean = 71.84, Standard Error = 6.73) when compared to those from Vrazov trg (Mean = 51.68, Standard Error = 9.33), showing the difference in DNA degradation between samples from previously mentioned sites (Figure 2). Descriptive statistics, test statistics, and tests for the normality of the distribution of the DNA yield and the degradation ratio can be seen in SM S2 (Tables S1–S3).

## 4. Discussion

While advancements in forensic and ancient DNA analysis continue to identify new genetic markers, the issue of sample preservation persists as a critical concern. During and after excavation, skeletal remains are subjected to an abrupt change in environmental conditions that profoundly impact the stability and integrity of bone DNA. This underscores the critical need for implementing effective preventive measures and ensuring proper storage protocols to maintain sample integrity. Such measures should mitigate DNA damage caused by spontaneous degradation and are particularly important in cases where DNA is already highly compromised, as seen in old and poorly preserved skeletal remains. As observed by Pruvost et al. [23], standard storage procedures can adversely affect DNA survival in fossil bones. To minimize DNA degradation, bone samples are commonly stored at −20 °C, as low temperature inhibits enzymatic activity and microbial proliferation [5,24]. However, recent studies have reported changes in bone crystallinity and increased DNA degradation associated with temperature fluctuations during freeze–thawing cycles [25]. Additionally, long-term freezer storage presents a significant financial burden for facilities storing human skeletal remains. Consequently, long-term storage at room temperature can be advantageous for facilities managing skeletal remains, but it is essential that a strict control of temperature and humidity is maintained to ensure the preservation of valuable genetic material.

When investigating the effect of storage at unregulated temperatures and humidity on the DNA yield and preservation in bone samples, our results revealed significant differences between freshly excavated samples and those stored at a museum depot for 12 years. A significant reduction in recovered DNA as well as an increase in DNA degradation can be observed for samples stored at unregulated temperature and humidity for 12 years. Since all samples in this study originated from the same geographical location and had equivalent post-mortem intervals, we can assume that the samples experienced no difference in other environmental factors affecting DNA survival, such as soil pH, external temperature, or hydrological conditions at the burial site. Our results, consistent with several other studies discussing the issue of storage of skeletal material [5,23], therefore suggest that storage conditions, especially unregulated temperature and humidity, detrimentally affect the amount and integrity of extracted DNA. Additionally, it is reasonable to conclude that the fluctuations in temperature and humidity are better shielded while the skeletal remains are still buried in the soil as it provides thermal insulation and regulated moisture levels. Indeed, soil represents a highly complex burial environment and includes many factors that can potentially influence and interfere with bone preservation [26]. The buffering effect most likely results in a stabilization of the microenvironment surrounding the remains and better DNA preservation in the case of freshly excavated skeletons in our study. Howes et al. reported similar findings in their study on bone degradation in different soil environments. They observed that while skeletal remains are still embedded in the soil, moisture content and temperature have a minimal impact on the preservation of organic components in bone tissue [26].

Based on our findings, we propose that archaeological skeletal remains be sampled for further genetic analysis immediately after excavation to avoid any DNA loss in the storage period. We advise that in cases where direct sampling after excavation is not possible, bone fragments are stored in museum depots following current scientific recommendations. According to our findings, unregulated temperatures and humidity pose a serious threat to DNA preservation in stored skeletal material. Building on previous research, which demonstrated a significant reduction in the DNA yield from bone fragments stored in freezers for 10 years [27], we join the recommendations of some of the world’s leading museums [8,9,10] regarding the optimal temperature and humidity for long-term storage of skeletal remains. Storage spaces should maintain a stable temperature between 16 and 20 °C, with relative humidity controlled within a range of 45–65%, as this range prevents mold growth, which occurs under excessively humid conditions while also avoiding bone cracking caused by low humidity [28]. In addition to humidity and temperature extremes, the fluctuation of both is known to enhance bone weathering and should be avoided [29,30]. To minimize these effects, the storage area should be protected against daily moderate temperature fluctuations as well as more extreme seasonal changes as both could contribute to the degradation of DNA in bone samples. Furthermore, other factors promoting DNA decay, such as exposure to UV light, should also be considered. Since UV light is known to induce DNA mutations and has more recently been shown to reduce bone density in exposed samples [31], it is crucial to store skeletal remains in environments with minimal or no sunlight exposure.

## 5. Conclusions

In this study, we aimed to investigate the impact of unregulated temperatures and humidity in long-term storage on DNA preservation in human skeletal material. To assess DNA preservation, the DNA yield and degree of DNA degradation were determined. According to our results, unregulated conditions in long-term storage detrimentally contribute to DNA degradation in stored bone samples. Considering the importance of reducing costs while sustaining DNA integrity in bone samples, we recommend that facilities storing human skeletal material regulate temperature and humidity levels within scientifically recommended ranges to ensure optimal DNA preservation.

**Key points**

1. Effective storage protocols for bone samples are needed to ensure optimal DNA preservation.
2. Freshly excavated petrous bones and petrous bones stored in a museum depot were sampled.
3. The DNA yield and degree of DNA degradation were compared for 186 bone samples.
4. Freshly excavated bones were found to have a higher DNA yield and a lower degree of DNA degradation.

## Data Availability

The original contributions presented in the study are included in the article/Supplementary Material, further inquiries can be directed to the corresponding author.

## Supplementary Materials

The following supporting information can be downloaded at https://www.mdpi.com/article/10.3390/genes16010078/s1. Supplementary Material S1—SM S1 Table S1: Sample characteristics (sample number, skeleton number, and number of graves), DNA quantity, and quality results (Auto, Deg, and Y target conveyed in ng DNA/μL, IPC shift, and Auto/Deg ratio) obtained using PowerQuant System (Promega) for petrous bones excavated from Vrazov trg archaeological site. Supplementary Material S1—SM S1 Table S2: Sample characteristics (sample number and skeleton number), DNA quantity, and quality results (Auto, Deg, and Y target conveyed in ng DNA/μL, IPC shift, and Auto/Deg ratio) obtained using PowerQuant System (Promega) for petrous bones excavated from Njegoševa archaeological site. Supplementary Material S2—SM S2 Table S3: Descriptive statistics of the degradation ratio (Auto/Deg) and DNA yield (ng DNA/g bone) for groups of bones from archaeological sites Njegoševa and Vrazov trg. Supplementary Material S2—SM S2 Table S4: Kolmogorov–Smirnov test (with Lilliefors significance correction) and Shapiro–Wilk test of normality for the Auto/Deg ratio and DNA yield (ng DNA/g bone) of petrous bones from the Njegoševa and Vrazov trg sites. Supplementary Material S2—SM S2 Table S5: Mann–Whitney U test, Wilcoxon W test, Z-test, and the Asymptotic Significance (2-tailed) for Auto/Deg ratio and DNA yield (ng DNA/g bone) with grouping variables: Njegoševa and Vrazov trg archaeological sites.
