# Supplementary material for: The Impact of Storage Conditions on DNA Preservation in Human Skeletal Remains: A Comparison of Freshly Excavated Samples and Those Stored for 12 Years in a Museum Depot
Source: Genes (Basel). 2025 Jan 11;16(1):78. doi: 10.3390/genes16010078 (PMC11764964; doi:10.3390/genes16010078)
Supplement: Supplementary file 1 [file genes-16-00078-s001.zip › SM 2.pdf]

**Supplementary Material S2**

**Table S3:** Descriptive statistics of the degradation ratio (Auto/Deg) and DNA yield (ng DNA/g bone), for groups of bones from archaeological sites Njegoševa and Vrazov trg.

| | 1=Njegoševa, 2=Vrazov trg | | | Statistic | Std. Error |
|---|---|---|---|---|---|
| Auto/Deg | 1 | Mean | | 71.844 | 6.732 |
| | | 95% Confidence Interval for Mean | Lower Bound | 58.488 | |
| | | | Upper Bound | 85.200 | |
| | | 5% Trimmed Mean | | 63.448 | |
| | | Median | | 52.740 | |
| | | Variance | | 4577.266 | |
| | | Std. Deviation | | 67.656 | |
| | | Minimum | | .33 | |
| | | Maximum | | 387.70 | |
| | | Range | | 387.37 | |
| | | Interquartile Range | | 68.60 | |
| | | Skewness | | 2.276 | .240 |
| | | Kurtosis | | 6.899 | .476 |
| | 2 | Mean | | 51.679 | 9.330 |
| | | 95% Confidence Interval for Mean | Lower Bound | 32.774 | |
| | | | Upper Bound | 70.583 | |
| | | 5% Trimmed Mean | | 43.506 | |
| | | Median | | 33.770 | |
| | | Variance | | 3307.850 | |
| | | Std. Deviation | | 57.514 | |
| | | Minimum | | .47 | |
| | | Maximum | | 332.57 | |
| | | Range | | 332.10 | |
| | | Interquartile Range | | 34.94 | |
| | | Skewness | | 3.509 | .383 |
| | | Kurtosis | | 15.315 | .750 |
| ngDNA/g bone | 1 | Mean | | 17.657 | 1.439 |
| | | 95% Confidence Interval for Mean | Lower Bound | 14.802 | |
| | | | Upper Bound | 20.513 | |
| | | 5% Trimmed Mean | | 15.909 | |
| | | Median | | 14.200 | |

| | | | | | |
|---|---|---|---|---|---|
| | | Variance | | 209.256 | |
| | | Std. Deviation | | 14.466 | |
| | | Minimum | | .40 | |
| | | Maximum | | 87.77 | |
| | | Range | | 87.37 | |
| | | Interquartile Range | | 14.17 | |
| | | Skewness | | 2.225 | .240 |
| | | Kurtosis | | 6.963 | .476 |
| | 2 | Mean | | 31.638 | 3.219 |
| | | 95% Confidence Interval for Mean | Lower Bound | 25.115 | |
| | | | Upper Bound | 38.161 | |
| | | 5% Trimmed Mean | | 30.327 | |
| | | Median | | 29.480 | |
| | | Variance | | 393.810 | |
| | | Std. Deviation | | 19.845 | |
| | | Minimum | | .01 | |
| | | Maximum | | 90.32 | |
| | | Range | | 90.31 | |
| | | Interquartile Range | | 21.30 | |
| | | Skewness | | 1.226 | .383 |
| | | Kurtosis | | 1.965 | .750 |

**Table S4:** Kolmogorov–Smirnov test (with Lilliefors significance correction) and Shapiro–Wilk test of normality for the Auto/Deg ratio and DNA yield (ng DNA/g bone) of petrous bones from the Njegoševa and Vrazov trg sites.

| | 1=Njegoševa, 2=Vrazov trg | Kolmogorov-Smirnov[a] | | | Shapiro-Wilk | | |
|---|---|---|---|---|---|---|---|
| | | Statistic | df | Sig. | Statistic | df | Sig. |
| Auto/Deg | 1 | .153 | 101 | <.001 | .786 | 101 | <.001 |
| | 2 | .261 | 38 | <.001 | .610 | 38 | <.001 |
| ngDNA/g bone | 1 | .140 | 101 | <.001 | .805 | 101 | <.001 |
| | 2 | .166 | 38 | .010 | .900 | 38 | .003 |

a. Lilliefors Significance Correction

**Table S5:** Mann-Whitney U test, Wilcoxon W test, Z-test and the Asymptotic Significance (2-tailed) for Auto/Deg ratio and DNA yield (ng DNA/g bone), with grouping variables: Njegoševa and Vrazov trg archaeological sites.

| | Auto/Deg | ngDNA/g bone |
|---|---|---|
| Mann-Whitney U | 1510.000 | 934.500 |

| | | |
|---|---|---|
| Wilcoxon W | 2251.000 | 6085.500 |
| Z | -1.933 | -4.653 |
| Asymp. Sig. (2-tailed) | .053 | <.001 |
